# Intravenous immunoglobulin frequency in multifocal motor neuropathy: a retrospective study of nerve conduction outcomes

**DOI:** 10.1186/s12883-026-05157-0

**Published:** 2026-07-10

**Authors:** Lili Zhou, Zizi He, Wei Fang, Hualan Yang, Xixi Sheng, Yake Meng, Xuzhen Zhu, Ruiming Wang, Hongfen Wang

**Affiliations:** 1 Department of Neurology, Hangzhou Hospital of Traditional Chinese Medicine, 453 Tiyu Stadium Road, Xihu District, Hangzhou, Zhejiang China; 2 Department of Neurology, Chinese People’s Liberation Army General Hospital, No. 28 Fuxing Road, Haidian District, Beijing, China; 3 Department of Neurology, Pudong Hospital of Fudan University, No. 2800 Gongwei Road, Shanghai, Huinan Town, Pudong New Area China; 4 Department of Orthopedics, Hangzhou Hospital of Traditional Chinese Medicine, 453 Tiyu Stadium Road, Xihu District, Hangzhou, Zhejiang China

**Keywords:** Multifocal motor neuropathy, Electrophysiology, Conduction block, Immunoglobulin, Treatment frequency

## Abstract

**Background:**

In resource-limited rural settings, patients with multifocal motor neuropathy (MMN) often present late due to limited access to neurophysiology services, by which time significant axonal damage may have occurred, rendering them less responsive to immunotherapy—yet this clinically subgroup is consistently underrepresented in randomized controlled trials. In this study, we aimed to examined the impact of intravenous immunoglobulin (IVIg) frequency on electrophysiological changes in such undertreated MMN patients.

**Methods:**

A retrospective analysis was conducted across three rural medical centers. Patients receiving > 2 IVIg cycles annually were classified as group A, others as group B. Nerve conduction was assessed at baseline, after the first treatment course, and at the final follow-up.

****Results**:**

Thirty-nine MMN patients initially identified, 11 patients with confirmed conduction block (CB) were included. 27 nerves exhibited definite CB, with 7 (25.9%) in the median nerve, 8 (29.6%) in the ulnar nerve, and 6 (18.5%) in both the tibial and peroneal nerves. The treatment intervals was 4.9 ± 1.8 (range, 2.6 to 8.0) months, The follow-up period was 17.2 ± 3.3 months (range, 12.0 to 21.0). 7 (63.6%) were in group A, while 4 patients (36.4%) were in group B. There was a clear improvement in mRS scores at the last follow-up compared to baseline (z = -2.795, *p* = 0.007). We compared the variations in distal compound muscle action potential (CMAP) amplitude at baseline and the last follow-up, after adjusting for baseline CMAP amplitude, it showed significantly greater improvements in distal CMAP amplitude in group A versus group B (F = 40.217, P < 0.001; B = 2.881, 95% CI 1.941 ~ 3.821, *P* < 0.001), with the model accounting for 71.5% of the variance (adjusted R²= 0.715). The number of CBs decreased from 17 to 14 in group A, with no change in group B (*p* > 0.05).

****Conclusions**:**

High frequency IVIg treatment may be associated with improved CMAP amplitude and mRS in undertreated MMN patients, suggesting potential benefits that require further validation.

**Supplementary Information:**

The online version contains supplementary material available at https://doi.org/10.1186/s12883-026-05157-0.

## Introduction

Multifocal motor neuropathy (MMN) is a chronic and progressive immune-mediated motor neuropathy [1–2]. Although considered very rare, MMN has a prevalence estimated at 0.6 to 2 per 100,000 population [2–3]. The onset of symptoms typically occurs between the ages of 20 and 75, the male-to-female ratio was 2.7:1 [1, 4]. MMN manifests as a gradual and asymmetrical weakness primarily affecting the upper extremities, and later involving the lower limbs. Initial symptoms often include finger flexor weakness and foot drop. The limb dysfunction contributes significantly to the impairment of quality of life in patients with MMN [1–3]. The presence of antibodies against the ganglioside GM1, a crucial component in maintaining the integrity of motor nerves, is an important characteristic of MMN [4–5]. Electromyography (EMG) is an essential tool for diagnosing MMN, and nerve conduction studies are characterized by persistent multifocal motor conduction block (CB) with or without secondary axonal damage [1, 2, 6–7]. The presence of CB is considered a significant hallmark of MMN, and it serves as a crucial indicator for assessing the therapeutic efficacy in MMN [1–2, 8].

Intravenous immunoglobulin (IVIg) currently stands as the primary treatment for MMN. Although more than 80% of patients experience improvement following treatment, only a small proportion achieve sustained remission, most patients require repeated IVIg infusions to prevent clinical deterioration [4, 8–9]. IVIg treatment, despite its proven efficacy, poses many challenges for many MMN patients due to its high cost and diagnostic delay, particularly among patients residing in rural areas [3, 10]. Previous studies have reported favorable responses to IVIg infusions administered at a dose of 0.4 g/kg/day over 5 consecutive days [4, 11–12]. However, the optimal treatment frequency have yet to be determined, especially in resource-limited rural regions where neurophysiological services and specialist expertise are scarce, patients with MMN frequently experience diagnostic delays. By the time a definitive diagnosis is established, secondary axonal degeneration may have already progressed substantially, rendering the condition less responsive to IVIg therapy. Thus, even when treatment is initiated, its efficacy may be attenuated, and the optimal frequency of IVIg administration in this specific population remains unclear. This patient subgroup—although routinely encountered in under-resourced clinical environments—remains conspicuously underrepresented in randomized controlled trial populations, representing a critical evidence gap in current practice [4, 9, 13]. In the study, we aimed to investigate the clinical characteristics and electrophysiological changes over time in undertreated MMN patients, with a specific focus on comparing the treatment effects of IVIg frequency on nerve conduction: more than two cycles per year versus fewer than two cycles.

## Materials and methods

### Patients and methods

A consecutive sample of medical records for patients aged ≥ 18 years old with a confirmed diagnosis of MMN was collected from three medical centers in rural areas of china between October, 2016, and November, 2022. Patients who met the following criteria were included: (i) Diagnosis of MMN based on the core clinical criteria and definite motor CB as proposed by the European Federation of Neurological Societies and the Peripheral Nerve Society in 2010 [2]; (ii) Stable symptoms and laboratory findings for a minimum of 6 months; (iii) Underwent at least one electromyography examination between the first and second IVIg therapeutic sessions; (iv) Received a fixed regimen of IVIg (0.4 g/kg for 5 days) without any dose adjustments throughout the entire treatment course; (v) Clinical functional scores, including the Overall Neuropathy Limitations Scale (ONLS) [14], modified Rankin scale (mRS) [15], and Hospital Anxiety and Depression Scale (HADS) [16] were recorded. Patients with any abnormal sensory nerve conduction velocities or sensory nerve action potential amplitudes were excluded from the study. Based on the frequency of IVIg treatment, patients were divided into two groups: (i) group A consisted of patients who received IVIg treatment for more than 2 cycles per year; (ii) group B included patients who received IVIg treatment for one or two cycles per year. To ensure data quality, we invited experts in neuromuscular diseases to review the medical records, particularly the EMG data. The data collection and collation were performed by different researchers, and the statisticians involved in the study were blinded to the research objectives. The study protocol was in accordance with the Declaration of Helsinki and approved by the ethics committee and research boards. The requirement for informed consent was waived by our institutional review board due to the retrospective nature of the study. All data were anonymized prior to analysis.

### Nerve conduction study

Nerve conduction was collected at three time points: baseline, after the first full course of treatment, and at the last follow-up. Nerve conduction studies were performed as follows. For the median nerve, segmental stimulation was delivered at the wrist, elbow, axilla, and Erb’s point. For the ulnar nerve, stimulation sites included the wrist, below the elbow, above the elbow, axilla, and Erb’s point. For the radial nerve, stimulation was applied at the elbow, radial groove, axilla, and Erb’s point. For the tibial nerve, stimulation was performed from the medial malleolus to the popliteal fossa. For the deep peroneal nerve, stimulation was applied from the ankle to below the fibular head. At each stimulation site, the following parameters of the evoked compound muscle action potential (CMAP) were recorded: amplitude, duration, area, latency, and waveform morphology. Distal and proximal stimulation were applied to record the CMAP amplitude, area, and duration. The amplitude ratio and area ratio were then calculated and compared with normal reference values to determine whether a significant decrease was present. Because data for the radial nerve were incomplete for a subset of patients, radial nerve-related parameters were excluded. We utilized the consensus criteria [2, 7], and definite CB is as the following criteria: (1) Proximal-to-distal CMAP amplitude or area ratio ≤ 50% for upper limb nerves and ≤ 60% for lower limb nerves; (2) Negative peak CMAP amplitude on stimulation of the distal part of the segment with motor CB must be > 20% of the lower limit of normal and > 1 mV; (3) Motor conduction velocity (MCV) reduction across the involved segment exceeds 30%; (4) Proximal CMAP duration prolongation ≥ 30% compared to distal CMAP duration. We specifically analyzed the number of CB, distal CMAP amplitude, and MCV only in nerves with definite CB.

### Statistical analysis

Employing SPSS 22.0 (IBM Corp, USA) for statistical analysis, measurement data are presented as mean ± standard deviation (Min, Max), or median (Min, Max). Categorical data are expressed as number of cases (n) and percentage (%), and group comparisons were carried out using Chi-square tests or Fisher’s exact test, as appropriate. The changes in distal CMAP amplitude and MCV from baseline to the last follow-up were compared between the two groups. For CMAP amplitude, an analysis of covariance (ANCOVA) was performed, with baseline CMAP amplitude values entered as covariates to adjust for potential baseline imbalances. Normality of the data was assessed using the Shapiro–Wilk test, and homogeneity of variances was examined using Levene’s test. As the MCV change data violated both parametric assumptions, between-group comparisons for MCV were conducted using the Mann–Whitney U test instead. A two-tailed *p* value < 0.05 was considered statistically significant. Graphical representation of the image data were conducted using GraphPad Prism.

## Results

### Clinical characteristics

**Fig. 1:**
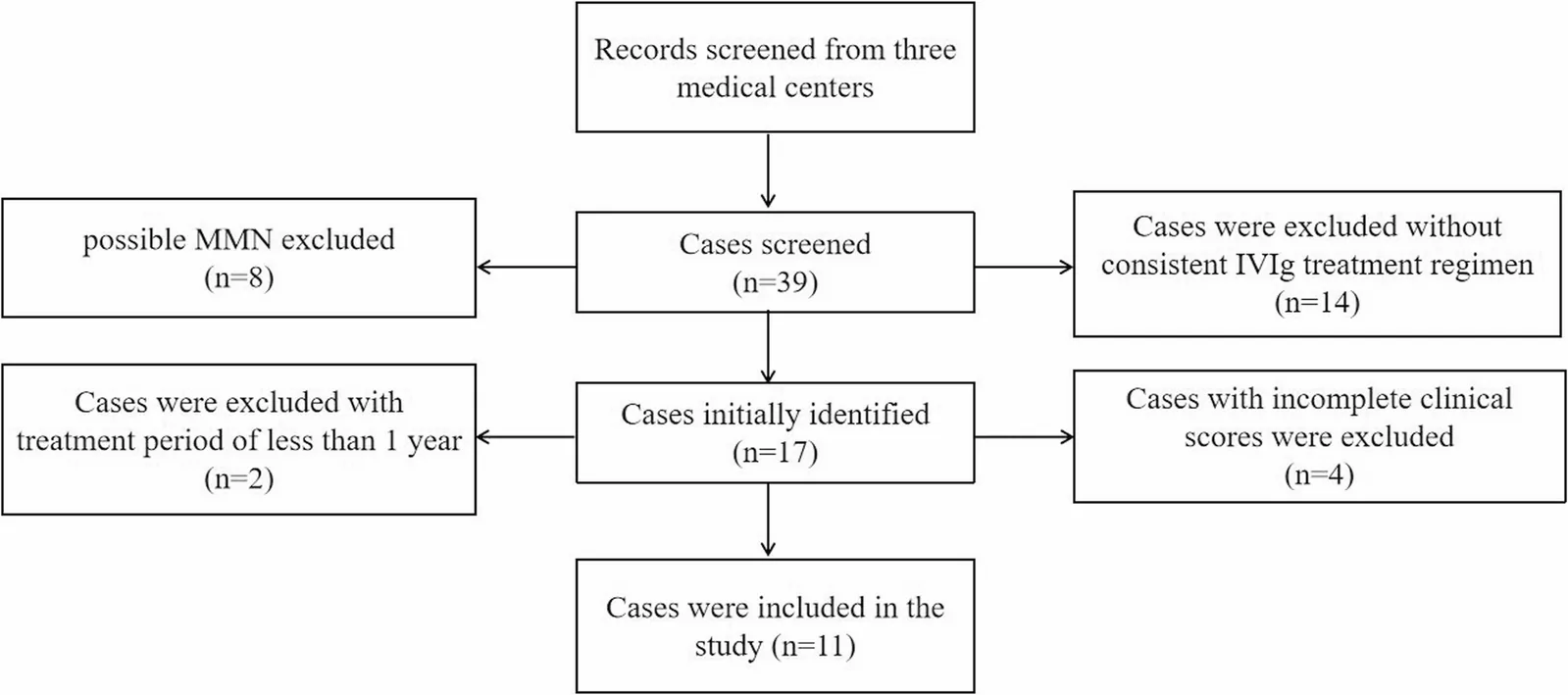
Flow chart of multifocal motor neuropathy cases identification

A total of 39 patients who met the diagnostic criteria for MMN were initially identified. Following discussions with experts, 8 patients with possible MMN were excluded from the study. Additionally, 14 patients who received varied IVIg doses or combined immunotherapy were excluded. Ultimately, a cohort of 11 patients with confirmed MMN was included (Fig. 1). Clinical characteristics of the included patients showed in Table 1. Initial muscle weakness was observed in a unilateral upper limb in 5 patients (45.5%), while 2 patients (18.2%) reported subjective sensory disorders. Hyporeflexia was present in 10 patients (90.9%), with 4 patients (36.4%) exhibiting more than two absent reflexes. Anti-GM1 (IgM) antibodies were detected in 8 patients (72.8%), and elevated cerebrospinal fluid (CSF) protein levels were found in 4 patients (36.4%). The treatment intervals was 4.9 ± 1.8 (range, 2.6 to 8.0) months, The follow-up period was 17.2 ± 3.3 months (range, 12.0 to 21.0). Among the patients, 7 (63.6%) were in group A, 4 patients (36.4%) were in group B. There was no significant decrease in ONLS scores between baseline and the last follow-up (z = -1.675, *p* = 0.116). However, there was a clear improvement in mRS scores at the last follow-up compared to baseline (z = -2.795, *p* = 0.007). The HADS anxiety and depression subscale scores showed no significant variations from baseline to the last follow-up (*p* > 0.05) (Table S1).

**Table 1:** Clinical and biological characteristics of 11 patients with MMN

	Total number of patients (*n* = 11)
Clinical characteristics	
Gender, male (%)	8 (72.8%)
Age at onset (y)	34.5 ± 12.1 (15.0, 51.0)
Disease duration (m)	18.0 (12.0, 60.0)*
Initial muscle weakness	
Unilateral upper limb	5 (45.5%)
Unilateral upper and lower limbs	2 (18.2%)
Unilateral lower limb	3 (27.3%)
Bilateral upper limbs	1 (9.1%)
Bilateral lower limbs	0
Subjective sensory disorders	2 (18.2%)
Tendon reflex	
Hyporeflexia^†^	10 (90.9%)
≤ one absent reflex	7 (63.6%)
≥ two absent reflex	4 (36.4%)
Laboratory data	
Anti-GM1 IgM antibodies (%)	8 (72.8%)
Elevated CSF protein	4 (36.4%)
Elevated creatine kinase	2 (18.2%)
Treatment intervals (m)	4.9 ± 1.8 (2.6, 8.0)
Group A (> 2 cycles/year)	7 (63.6%)
Group B (1–2 cycles/year)	4 (36.4%)
Follow-up period (m)	17.2 ± 3.3 (12.0, 21.0)

Data were n (%) or mean ± SD (Min, Max)

*y *years, *m* months. *MMN *multifocal motor neuropathy, *CSF *cerebrospinal fluid

^†^one patient had normal reflex, *Median (Min, Max)

### Motor nerve conduction study

Motor nerve conduction study data with adjustment for baseline covariates was pre-specified as the primary analysis by experts from different centers. Motor nerve conduction examinations were conducted in all 11 patients, involving bilateral median, ulnar, tibial, and peroneal nerves. Among these, 27 nerves exhibited definite CB, with 7 (25.9%) in the median nerve, 8 (29.6%) in the ulnar nerve, and 6 (18.5%) in both the tibial and peroneal nerves (Table 2, showed the nerve distribution of CBs). The number of conduction blocks (CBs) in group A was 17 in baseline and 14 at the last follow up, there was no decreased in the number of CBs in group B in baseline and the last follow up, and the differences between the two groups were not statistically significant (all *p* > 0.05) (Table 3, showed the number of CBs).

**Table 2:** Nerve distribution of CBs in 11 patients included 27 nerves with MMN

Nerve distribution of CBs	Number of CBs		
	Group A (> 2 cycles/year) (*n* = 7)	Group B (1–2 cycles/year) (*n* = 4)	Total (*n* = 11)
Median	4	3	7 (25.9%)
Ulnar	5	3	8 (29.6%)
Tibial	4	2	6 (18.5%)
Peroneal	4	2	6 (18.5%)

Data were n (%)

*CBs *conduction blocks, *n *number of patients, *MMN *multifocal motor neuropathy

**Table 3:** The number of CBs in 11 patients included 27 nerves

Group	Baseline			At last follow-up			*X*²	*P*
	Number of CBs	Number of non-CBs	Total number of detected nerve fibers	Number of CBs	Number of non-CBs	Total number of detected nerve fibers		
Group A (> 2 cycles/year) (*n* = 7)	17	36	53	14*	42	56	0.670	0.413
Group B (1–2 cycles/year) (*n* = 4)	10	20	30	10	18	28	0.036	0.849
*X²*	0.014			1.050				
*P*	0.906			0.306				

The comparisons between the two groups were all conducted using Chi-square Test. *P* value less than 0.05 is considered statistically significant

*CBs *conduction blocks, *n *number of patients, *x² *Chi-square value

*including one patient had a small distal CMAP amplitude in ulnar nerve and in the following distal CMAP amplitude was absent

**Fig. 2:**
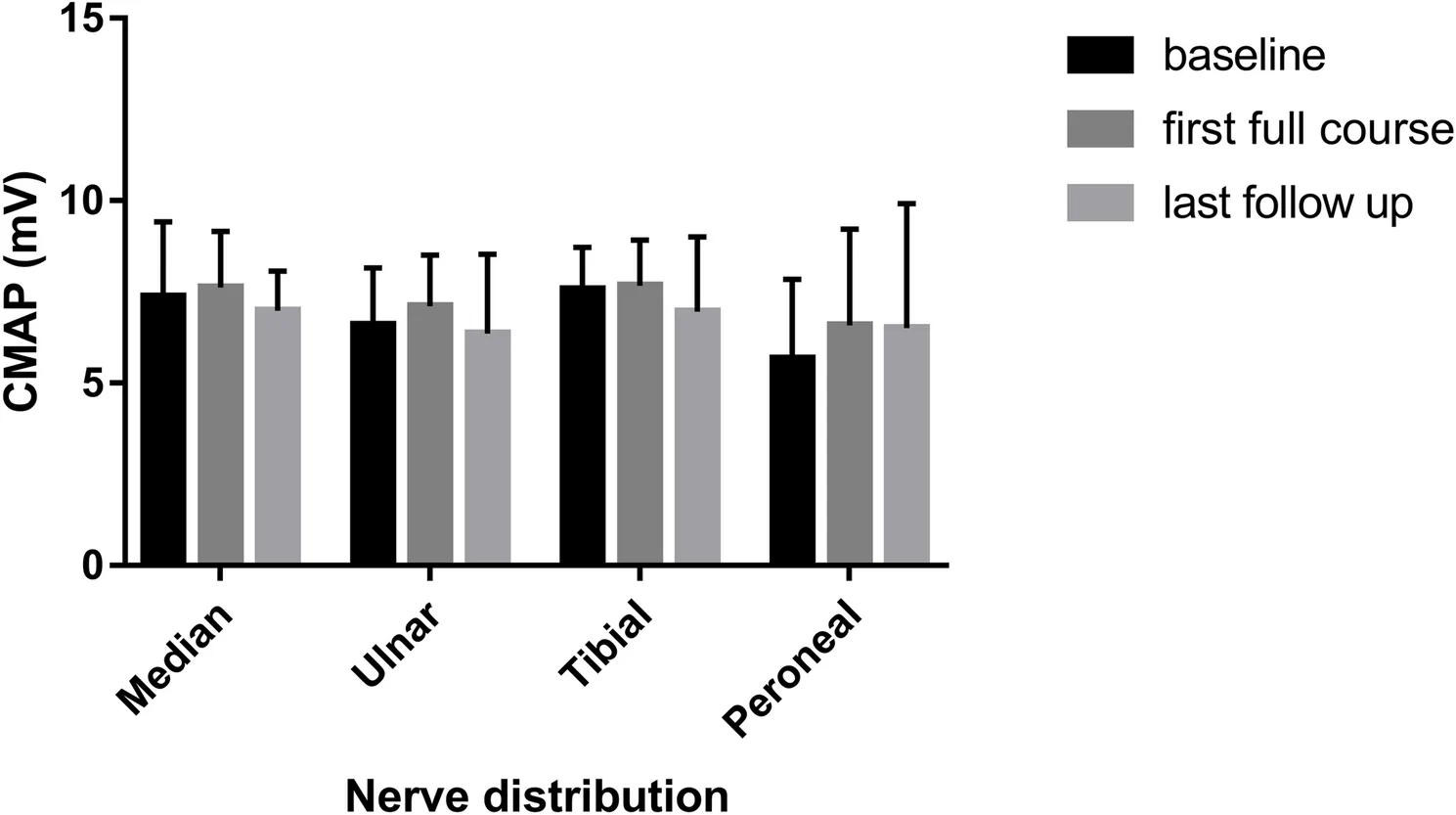
Distal CMAP amplitude on the different nerves among the baseline, the first full course and the last follow-up in 11 patients with MMN. CMAP: compound muscle action potential. MMN: multifocal motor neuropathy

**Fig. 3:**
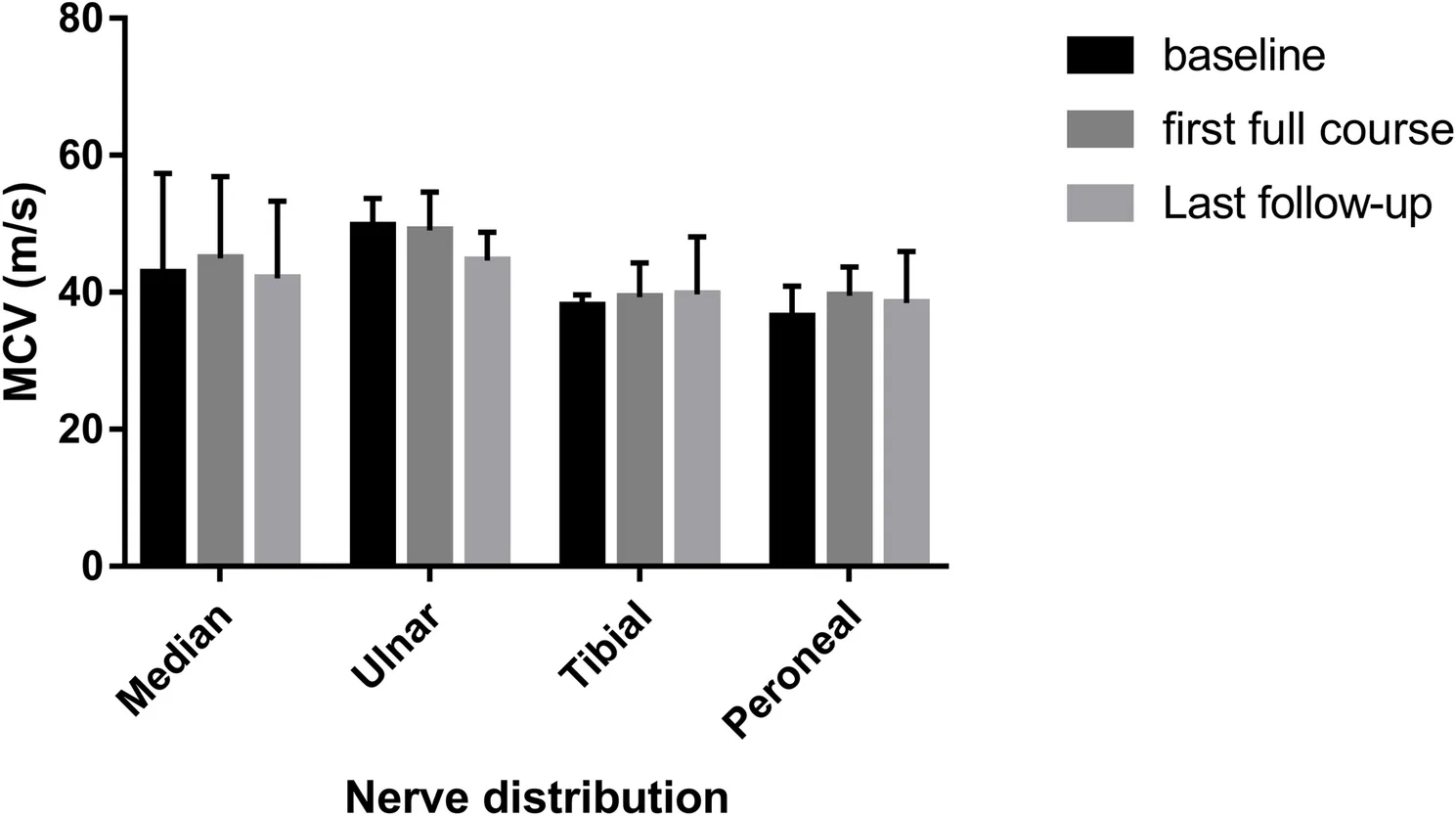
MCV on the different nerves among the baseline, the first full course and the last follow-up in 11 patients with MMN. MCV: motor nerve conduction velocity. MMN: multifocal motor neuropathy

It was showed that group A consisted of 7 patients with a total of 16 nerves, while group B included 4 patients with a total of 10 nerves. The variations in distal CMAP amplitude and MCV in nerves with CB across all patients at baseline, after the first full course of treatment, and at the last follow-up are depicted in Figs. 2 and 3. We compared the changes in distal CMAP amplitude and MCV at baseline and the last follow-up, it revealed a significant group difference in distal CMAP amplitude (F = 40.217, *P*  < 0.001) after adjustment for baseline CMAP amplitude (F = 33.263, *P * < 0.001), the variations were significantly more prominent in group A (B = 2.881, 95%CI 1.941 ~ 3.821, *P* < 0.001), which exhibited substantially better CMAP recovery at the final follow-up relative to group B, the overall model was significant (F = 32.383, *P* < 0.001, adjusted R^2^ = 0.715); and a better MCV recovery was also observed at the last follow-up in group A (z = -4.217, *p* < 0.001) (Table 4).

**Table 4:** The changes of distal CMAP amplitude and MCV between the baseline and the last follow-up in nerves with definite CB with MMN

Parameter	Group A (> 2 cycles/year) (*n* = 16†)	Group B (1–2 cycles/year) (*n* = 10)	F/Z	*P*
Variations of distal CMAP amplitude (mV)	0.95 ± 1.04	-2.14 ± 1.24	40.217*	< 0.001
Variations of MCV (m/s)	5.14 ± 4.50	-9.60 (-12.93, -7.58)	-4.217**	< 0.001

Data were mean ± SD or median (Q1, Q3)

*CB *conduction block, *n* number of CB, *MMN *multifocal motor neuropathy, *CMAP *compound muscle action potential (CMAP), *MCV *motor nerve conduction velocity, *Q1 *the first quartile, *Q3 *the third quartile

* P *value less than 0.05 is considered statistically significant

*Analysis of covariance, **Mann-Whitney U test,  †one patient had a small distal CMAP amplitude in ulnar nerve and in the following distal CMAP amplitude was absent, so at the last follow-up, only 26 nerves were included

## Discussion

MMN is a very rare disease. In the study, we investigated whether higher frequency of IVIg cycles—specifically comparing more than two cycles per year against two or fewer—improves nerve conduction and clinical outcomes in patients with definite MMN. The data comes from rural medical centers and this kind of population is often underrepresented in clinical trials. Our findings underscore a clinically meaningful implication for the management of MMN, particularly for patients frequently encountered in resource-limited settings.

In most patients with MMN, poor hand dexterity and fine motor skills often significantly impair social and professional functioning, while distal lower extremity weakness may lead to imbalance and falls. In our study, 5 (45.5%) patients were initial unilateral upper limb weakness, 7 (25.9%) were median nerves, 8 (29.6%) were ulnar nerves, 6 (18.5%) in both the tibial and peroneal nerves. In the literature, the most commonly affected nerves were the median and ulnar nerves, whereas radial nerve involvement is relatively uncommon [2, 3, 17]. In our retrospective analysis, we observed that radial nerve data were missing for a subset of patients. To ensure comparability and consistency between study groups, we excluded the incomplete radial nerve data from the present analysis. In future studies, we will enroll a larger cohort and include complete radial nerve data to enable more comprehensive analysis.

A high-positive titer of anti-GM1 antibodies may be of diagnostic value, as demonstrated in our study where anti-ganglioside GM1 IgM antibodies were detectable in 8 of 11 patients (72.8%). The reported prevalence of anti-GM1 IgM antibodies in MMN varies substantially across studies, ranging from 0% to 100% [18–19]. This wide variation is largely attributed to differences in enzyme-linked immunosorbent assay (ELISA) methodologies, including the use of detergents, serum incubation conditions, and cut-off values, our detection rate of 72.8% is consistent with studies employing optimized assay conditions. We had aimed to assess the association between persistent serum anti-GM1 IgM positivity and treatment intervals or remission. However, the inability to collect sufficient serial antibody measurements during the follow-up period prevented this analysis. Studies had found no significant difference in response to IVIg treatment between patients who are seropositive and those who are seronegative for anti-GM1 IgM antibodies [5, 20].

IVIg is a key therapy for MMN. IVIg treatment has been shown to reduce the occurrence of CB [21–22]. However, the improvement in CB is not easily observable or predictable. Although some research had suggested that dosing intervals shorter than four weeks could mitigate fluctuations in symptoms, the study did not include nerve conduction studies [23]. In our study, we followed up the motor nerve conduction parameters and found that although IVIg treatment led to an initial decrease in the number of CBs in all patients, as ongoing clinical progression, at the final follow-up, the number of CBs was not significantly improved compared to baseline. Although this difference of CBs between two groups after IVIg treatment did not reach statistical significance, the number of CBs in group A was 17 in baseline and 14 at the last follow up, while there was no decreased in the number of CBs in group B in baseline and the last follow up. CB in MMN may be absent, transient, or located in proximal nerve segments that are difficult to assess, additional nerve conduction parameters beyond CB may hold clinical significance [24]. In our study, group A exhibited significantly better CMAP and MCV recovery at the final follow-up relative to group B. The maintenance treatment of IVIg had demonstrated a beneficial long-term effect on nerve conduction variables in nerves with CB, IVIg has demonstrated benefits in slowing axonal loss, it does not completely arrest disease progression, with serial nerve conduction studies often showing persistent CB despite clinical stabilization or improvement [10, 13, 21, 22]. To our knowledge, this is the first real-world study to report an association between high-frequency IVIg induction and CMAP amplitude improvement in undertreated MMN patients. Although our study did not demonstrate a superior reduction in CB following high-frequency IVIg treatment, we observed significant improvements in CMAP amplitude. The CMAP amplitude changes often reflects the reversal of distal CBs. In line with this interpretation, a recent investigation by Doneddu et al. revealed that 71% of MMN patients show nerve conduction abnormalities extending beyond conventional CB [24], with a significantly more frequency than lower motor neuron disease or axonal polyneuropathies. These findings align with previous studies suggesting that electrophysiological abnormalities characteristic of demyelinating and paranodal neuropathies form part of the MMN electrophysiological profile [25–27]. Collectively, this evidence supports the clinical value of comparing MCV and CMAP data in our study, as these parameters capture meaningful treatment-related changes beyond the traditional reliance on CB assessment alone.

Although the Rasch-built Overall Disability Scale for Multifocal Motor Neuropathy (MMN-RODS) has been developed for MMN, we chose the ONLS to assess functional disability in our cohort. The MMN-RODS, while sensitive, requires prospective collection of 25 items and Rasch-based transformation, making it difficult to apply to historical data. In contrast, the ONLS is brief, interview-based, and can be reliably scored from clinical records [14, 28]. In our study, the ONLS did not show significant improvement following high-frequency IVIg treatment, whereas the mRS—a global disability scale—did. This apparent discrepancy warrants careful interpretation. The ONLS is a neuropathy-specific scale that is particularly sensitive to fine upper limb functional changes, whereas the mRS places greater emphasis on ambulation and global independence [15]. The differential responsiveness we observed may reflect the pattern of recovery in MMN: lower limb strength and walking ability (captured by mRS) may improve earlier or more completely following IVIg, whereas distal upper limb fine motor function (captured by the ONLS upper limb component) often recovers more slowly or incompletely, particularly in patients with prolonged disease duration and established axonal loss prior to treatment. This interpretation is consistent with our electrophysiological findings, which showed CMAP improvements (suggesting reversal of distal CBs) but did not achieve full normalization. Thus, while high-frequency IVIg conferred meaningful benefits in global disability and muscle strength, the ONLS-by capturing residual deficits in fine hand function-may reflect a domain that requires more prolonged or intensive rehabilitation. Future studies with longer follow-up are needed to determine whether ONLS improvements emerge after extended treatment. CB is not a reliable parameter for assessing IVIg response, as it may be absent, transient, or located in proximal nerve segments inaccessible to routine nerve conduction studies. Instead, validated functional tools such as the MMN-RODS and hand grip strength are more clinically meaningful for longitudinal follow-up. While our study did not include the MMN-RODS, we used mRS, ONLS as functional endpoints. Electrophysiological parameters were included only as exploratory endpoints. Future prospective studies should prioritize MMN-specific functional scales and hand grip strength as primary outcome measures. Depression rose from 8% to 25%, and longer disease duration/disability correlate with poorer mental health [29–31]. The HADS screened for mood disturbances in MMN, despite no significant electrophysiological correlations, these descriptive data underscore MMN’s non-motor burden.

Our study has several limitations. Firstly, the sample size was relatively small, as well as the detected number of CBs. Given that CB can also be present during the early course of other peripheral neuropathies, including Guillain-Barré syndrome (GBS) and chronic inflammatory demyelinating polyneuropathy (CIDP), stringent selection criteria were implemented to reduce the misdiagnosis rate. Therefore, only cases with definite CB were enrolled, and probable partial CB cases were excluded, following the stringent exclusion of all suspected cases. We believe that our results are valid in patients with definite MMN. All patients received a fixed regimen of IVIg without any dose adjustments throughout the treatment course. In nerve conduction study, we only collected the motor nerve parameters with definite CB, and at the last follow up, as ongoing clinical progression, reliable CB had particularly challenging when the amplitude of the distal CMAP was less than 1mV. Secondly, the lack of predefined or randomized IVIg treatment frequency is a significant limitation. Factors such as baseline disease severity, individual clinical progression trajectories, and socioeconomic limitations—including financial barriers and geographic access to infusion centers—may have independently influenced both the assigned treatment intervals and the observed clinical outcomes. In rural regions with limited access to neurophysiology services and specialists, patients often present late. As a result, by the time treatment is initiated, significant secondary axonal degeneration may have already occurred, which is less responsive to immunotherapy. In clinical practice, MMN is typically treated every 2 to 5 weeks [2, 23, 32]. Our findings may not be directly generalizable to patients receiving optimal maintenance therapy. However, we believe our study provides valuable evidence for the management of undertreated MMN patients—a population frequently encountered in resource-limited settings but typically excluded from randomized controlled trials, the between—group comparisons presented in this study should be interpreted as exploratory and hypothesis-generating, not as evidence of causal treatment effects. Prospective randomized controlled trials are needed to definitively compare different IVIg regimens in MMN. Thirdly, this study is the lack of needle EMG data, and correlation analyses between clinical scores and the degree of axonal damage were not performed. As reported by Vucic et al., both CB and axonal degeneration correlate significantly with muscle weakness in MMN [33]. Additionally, one study demonstrated that decreased motor unit number index is the most important determinant of permanent weakness and disability [34]. Due to the limited sample size in our study and the retrospective design with missing radial nerve data in some patients, needle electrode jitter analysis had not been performed as part of the original study, we were unable to perform reliable correlation analyses. Lastly, our study used the mRS as the primary functional outcome measure, which assesses global disability but does not provide muscle-specific strength data. Consequently, we cannot identify which individual muscle groups contributed to functional improvement. Future studies should incorporate muscle-specific strength assessments (e.g., MRC sum scores or hand grip dynamometry) to provide more granular information on treatment response. And the relatively small sample size, formal correlation analyses between clinical scores (such as mRS, RODS, MRC and ONLS) and electrophysiological parameters were not statistically feasible. Larger prospective studies are needed to explore these potential relationships. Our research team plans to discuss and integrate this standard into the design of future studies. Notwithstanding these limitations, our findings underscore a clinically meaningful implication for the management of MMN, particularly for patients frequently encountered in resource-limited settings.

## Conclusion

In conclusion, this retrospective study of a small cohort of undertreated MMN patients found that high-frequency IVIg therapy was associated with improvements in CMAP amplitude, and mRS. Our findings underscore a clinically meaningful implication for the management of MMN, particularly for patients frequently encountered in resource-limited settings. However, due to the non-randomized design, small sample size, and potential for confounding, these findings are hypothesis-generating only. They do not provide a basis for changing current clinical practice. Larger, prospective, randomized controlled trials are needed to determine whether high-frequency IVIg regimens confer meaningful advantages over standard maintenance protocols and, if so, which regimen is optimal. Until such evidence is available, treatment decisions should continue to follow existing guideline-based recommendations.

## Supplementary Information

Supplementary Material 1: Figure S1. The variations of distal CMAP amplitude in patients with MMN between short and long treatment period. b: baseline. f: first full course. l: last follow-up. Median, ulnar, tibial, peroneal nerves were respectively showed in green color, red color, blue color, orange color. short treatment period: received IVIg treatment for more than 2 cycles per year. long treatment period: received IVIg treatment for less than 2 cycles per year. CMAP: compound muscle action potential. MMN: multifocal motor neuropathy.

Supplementary Material 2: Figure S2. The variations of distal MCV in patients with MMN between short and long treatment period. b: baseline. f: first full course. l: last follow-up. Median, ulnar, tibial, peroneal nerves were respectively showed in green color, red color, blue color, orange color. short treatment period: received IVIg treatment for more than 2 cycles per year. long treatment period: received IVIg treatment for less than 2 cycles per year. MCV: motor nerve conduction velocity. MMN: multifocal motor neuropathy.

Supplementary Material 3 .

## Data Availability

The data that support the findings of this study are available from the corresponding author upon reasonable request.

## Abbreviations

MMN: Multifocal motor neuropathy
IVIg: Intravenous immunoglobulin
CB: Conduction block
CMAP: Compound muscle action potential
MCV: Motor nerve conduction velocity
ONLS: The Overall Neuropathy Limitations Scale
mRS: Modified Rankin scale
HADS: Hospital Anxiety and Depression Scale
